# Multiple drivers of ecological change in Arctic lakes and ponds

**DOI:** 10.1371/journal.pone.0254257

**Published:** 2021-07-30

**Authors:** Konrad Gajewski

**Affiliations:** Laboratory for Paleoclimatology & Climatology, Department of Geography, Environment & Geomatics, University of Ottawa, Ottawa, Ontario Canada; Universidade de Vigo, SPAIN

## Abstract

Griffiths et al. (2017) analyzed several ponds and lakes from the Cape Herschel region of Ellesmere Island in order to “…*explicitly examine the role of ice cover as the dominant driver of diatom assemblage change*…”. I reanalyze their data and suggest that their classification scheme, that they propose is due to differences in ice cover seasonality (“warm”, “cool”, “cold”, and “oasis”), is confounded with other morphological and chemical variables that better explain the differences between the groups. The “cold” sites are the deepest (lakes) and differ from the small, shallow ponds that occasionally dry, which would therefore have different diatom assemblages and histories. The “oasis” sites are nutrient enriched and probably have more stable water supplies, thereby enabling an aquatic flora providing habitats for diatoms. A key part of their interpretation is that “warm” sites have responded more rapidly to recent climate change than “cold” or “cool” sites, but their chronologies do not allow for such a conclusion. There is no clear difference between “cool” and “warm” sites, and problems in dating the sequences means inferences about their histories are not supported by data. Their results, which are restricted to the past century, are contradicted by a Holocene sequence from the region.

## Introduction

Griffiths et al. (2017) [1] analyzed several ponds and lakes from the Cape Herschel region of Ellesmere Island. Their stated purpose was to “*explicitly examine the role of ice cover as the dominant driver of diatom assemblage change*…” [1]. They took short sediment cores from 10 lakes and ponds in the region that they divide into four classes–“cold”, “cool”, “warm” and “oasis”. They hypothesized that changes in the diatom assemblages should occur earlier in warmer sites than in “cool” sites, that “cold” sites should show little change, and that “oasis” sites would have been more diverse for a longer period of time ([1]; pg 3). They concluded that their predictions held up [1].

However, their conclusions, especially as articulated in the title and abstract, do not follow from the data. In several places in the paper, the authors place caveats on their interpretation, although they underestimate the severity of these issues. There are three main issues with the paper:

1. Griffiths et al. [1] divide their sites into four classes—“warm”, “cool”, “cold”, and “oasis”- and this is the basis of all interpretation, however they have insufficient data on ice cover, and do not consider other factors, such as lake depth and nutrients.
2. Problems with their chronologies and lack of dating in some cores make it difficult to test hypotheses about the timing of changes in the diatom assemblages.
3. The interpretation of changes in the diatoms in these short cores is contradicted by a longer sediment sequence from the same area. In addition, diagenesis or mixing of the uppermost sediments makes it difficult to draw conclusions from such extremely short sediment sequences.

## Methods

Data for this study were provided by the authors, as per the editorial policy of PLoS-One, and the data files referred to below were taken from [1] (doi: 10.5061/dryad.g7h7n). Data for Fig 2 are from (http://www.lpc.uottawa.ca/data/pcsp/pcspdata.html). Analysis and graphing was done in C2 [2] or R [3]. A principal components analysis of the limnological data from [1] was performed on a correlation matrix using the package vegan (rda) [4].

## Results and discussion

### 1) Griffiths et al. (2017) divide their sites into four classes—“warm”, “cool”, “cold”, and “oasis”- and this is the basis of all interpretation, however this classification is not supported by data

**Fig 1:**
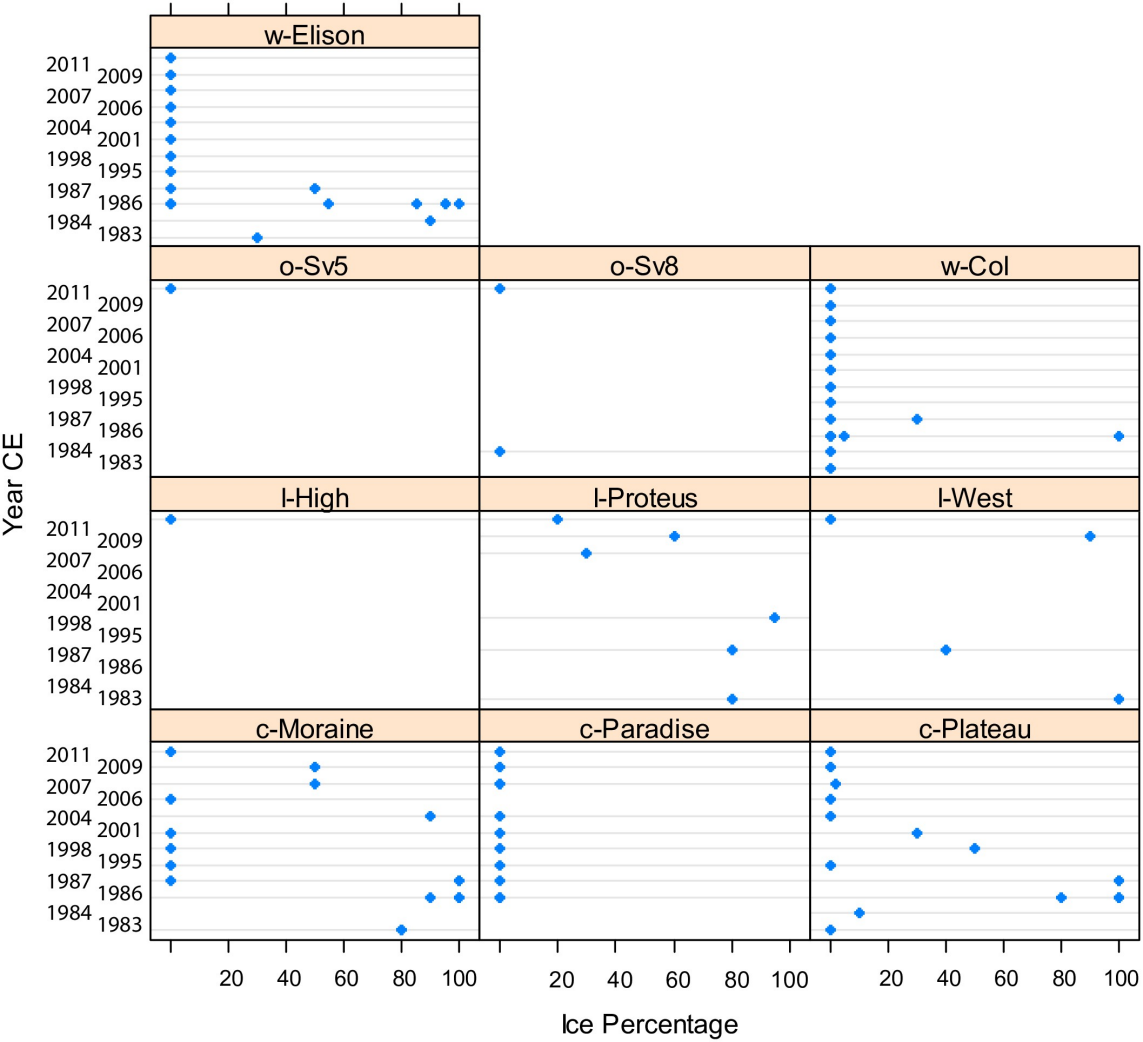
Dotplot of the data in Griffiths et al. (2017); their document S1 Data. For each year with data (years 1–12), the ice percentage recorded on the day of sampling is plotted. Note that some sites had more than one observation on a different day of the year. “c” are “cool” sites, “l” are “cold” sites, “o” are “oasis” sites and “w” are “warm” sites as classified by Griffiths et al. (2017).

Griffiths et al. [1] state that the: “*Rationale for group divisions is based on nearly 30 years of sampling…”* [1], however the lakes were only visited one to 12 years intermittently over a 30-year period. The classification into “Warm”, “Cool”, “Cold”, and “Oasis” is based on observations of the presence or absence of ice cover on one or several days of the year in mid-summer, or more times during the summer on only rare exceptions ([1], their S1 Table). Their rationale for division into these classes is summarized in their Table 1 and elaborated in their Site Description section. This division is based on anecdotal information such as snow cover in the catchment or observations made during helicopter surveys. Since these classes are the basis of all further analysis, they should be based on objective and reproducible data, rather than assumptions about the microclimate. For example, they could place data loggers in all sites for several years, or use repeat photography to quantify the growing season length. It is difficult to clearly separate “cold” and “cool” sites based on these data as both contain a number of years with ice cover on one day of the year (Fig 1). The “cool” site Paradise Lake looks like “warm” sites Col Pond and Elison Lake. But they provide no data on the length of the ice-cover or the ice-free season.

**Table 1:** Available temperature data from Sverdrup Pass (four sites) and Herschel (four sites).

Month	Sverdrup		Herschel	
	Number of measures	Number of years	Number of measures	Number of years
Apr	92	2		
May	14	2	401	12
Jun	97	3	497	11
Jul	288	6	325	8
Aug	168	8	160	6
Sept	18	1		

Data are twice daily (in most cases) measurements of temperature sent to the Resolute Bay base by observers in the field [20].

Further, the use of one or several days of the year as the index of length of growing season is problematic. Hobbie [5] describes the ice melt process on lakes, which goes through a series of eight stages; briefly, snow melts, so water accumulates on top of the lake ice, then the ice becomes porous, so the water drains. The ice candles, an ice moat develops and during this time “…*the ice floats free of the shore and portions may crack and be moved about by the wind*” [5, p. 69], disturbing the sediment in areas less than ~2 m deep. This phase is followed by breakup. As the candles break apart, they tip on their sides, so he notes that “*a lake 80% covered with ice can become ice free in 24 hours*.” [5, p. 69]. Therefore one midsummer observation of ice cover represents little about the seasonal cycle. This applies to lakes or deep ponds; in the very shallow systems studied in [1], a few centimeters of the sediment itself thaw and refreeze every year, and the time it takes ice cover to melt depends on the depth of the water and snow cover. At all sites, the continual disturbance has nothing to do with climate variations, as it would happen every year.

The “oasis” sites are located inland from the other three groups, and they may therefore be expected to be warmer, although the increase in altitude may negate this [6]. Labine [7] detailed the climate of the Alexandra Fiord lowland, a coastal oasis located 35 km from Cape Herschel, and illustrated many factors that must be considered to explain an oasis, including synoptic situation, local albedo, or distance from coast. Labine maintained automatic weather stations in five sites for five years. Temperatures varied among the different areas of the oasis and greatly from year to year. Soil temperatures (and presumably water temperatures in small ponds) depended greatly on their colour which affected absorption of radiation, and indeed, were greater in non-oasis than in “oasis” sites. The large variability shown in [7] makes it difficult to infer micro- or mesoclimate temperatures from small scale maps or studies which are based on few data or from casual observations, especially to obtain the precision needed to test the hypotheses of Griffiths et al. [1].

Vegetation in the Arctic is greatly dependent on available moisture, which partially determines nutrient availability, as well as on temperature. The “oasis” sites are near glaciers, whose outflow or seepage in summer could presumably supply moisture not available to other sites, and depending on the local topography, availability of moisture would lead to the denser vegetation. The kind of bedrock could also contribute to differences in plant production [8, 9]. To extrapolate from the vegetation to ice cover on the lakes (sampled one or two times) is not properly justified.

Griffiths et al. [1] suggest the “oasis” sites “*historically had elongated ice-free periods in summer*, *prior to anthropogenic warming*” ([1] page 3 and Table 2 caption), although there is no evidence for this assertion and indeed, the evidence suggests the opposite. The SV sites are located near outlet glaciers and there are ice caps to the North and South of the pass; these expanded during cool periods such as during the Little Ice Age [10]. Other evidence Griffiths et al. [1] present is that the area was deglaciated 5000–6000 years ago and indigenous people have traversed the area for many years ([1] their Table 1). However, the entire region, including their other sites, has been deglaciated for thousands of years [11; [1] reference 32], and supported human populations [12; [1] ref 24]. They provide several references in support of their assertion that these had the longest ice-free season, however these references do not provide data that this area has a longer or shorter ice-cover season than the regions of the other classes. Levesque [13; [1] ref 33] is about polar desert communities and not oasis sites. Elster et al. [14; [1] ref 34] present no environmental data and they mention that the dense associations of vascular plants are associated with streams from the glaciers. They further say that during the Little Ice Age (CE1450-1850), uplands were covered by more extensive snow, suggesting the area was colder than today. Henry et al. [15; their ref 35) is based primarily on a helicopter survey of Sverdrup Pass as well as limited vegetation survey (but no temperature data) and who also point out that “*…relatively well-vegetated areas … occur in moist coastal lowlands*”. The presence and population dynamics of the muskox, mentioned by Griffiths et al. [1] as evidence of warmth, is maintained by migration from the Fosheim Peninsula [15]. Finally, their reference 37 [16] refers to Lake Hazen, located 300 km north of Sverdrup Pass. Although speculating on the reasons for Hazen’s oasis status, this paper also presents no climate data; in any event, this paper is of no relevance here as the geographic situation is completely different.

**Table 2:** Summary of chronology information for sites in Griffiths et al. (2017).

				^210^Pb					^14^C				
Group	Pond	Lake Water Depth	Depth (top of interval) where unsupp>supp	Age of deepest estimate	Range of 1 std of last level	2nd-deepest age estimate	Depth (top of interval) of Cs peak	Sample Depth	Median age	1 std	Lowest depth analyzed for diatoms	# levels dated	# levels extrap-olated
		m	cm	yrCE	yrCE	yrCE	cm	cm	cal BP	cal BP	cm	#	#
“Cool”	Moraine	0.5	8	1880	1730–1990	1900	2	-	-	-	10	24	2
	Paradise	1.8	3	1900	1710–1970	1980	0	-	-	-	9	13	23
	Plateau	0.3	3	1840	1440–2040	1910	0	-	-	-	5	12	3
“Cold”	High	4.0	1	1920	1200–2023	1980	0	-	-	-	5	5	15
	Proteus	6.9	2	1910	1400–2020	1960	0	-	-	-	7	9	18
	West	12.1	10	1890	1880–1900	1900	1	-	-	-	24	41	21
“Oasis”	SV5	0.5	-	-	-	-	-	9.5	2322	2310–2340	10	0	16
	SV8	0.3	4	1940	1930–1940	1970	1	10	3050	3000–2070	9	10	9
“Warm”	Col	0.5	4.0/0.5*	-	-	-	-	-	-	-	4.5	0	16
	Elison	1.5	3.0/2.3*	-	-	-	-	45	4260	4250–4420	4.5	0	15

Depths are at the top of the interval.

* the first number is the depth reported in Douglas et al. (1994), and the second is Griffiths et al. (2017)

There are other studies not mentioned by Griffiths et al. [1] that do discuss Sverdrup Pass or other oases in more detail. Levesque et al. [8] studied vegetation of the Sverdrup Pass and presented five years of climate data. Temperatures were above zero typically through June-August, although with days with mean temperatures below zero during this period. They also show large interannual variability in the weather, suggesting that one or two years of observation may not be representative of the longer-term values. They emphasize the importance of bedrock type on plant growth. Bergeron and Svoboda [17] emphasize soil moisture as a factor explaining the increased vegetation density of Sverdrup Pass in relation to surrounding uplands. They state that July temperatures are high (“*>5°C*”), although details are not provided. However July temperature normals (1981–2010) are 5.1°C at Nanasivik, 4.5°C at Resolute Bay and 3.4°C at Alert, all polar deserts, as well as 6.1°C for Eureka and 6.6°C for Pond Inlet (data from http://climate.weather.gc.ca/). Koizumi [9] suggests that the reason for the presence of the “oasis” is, in fact, bedrock type, and in a study of muskoxen in Sverdrup Pass, Raillard and Svoboda [18] concluded *“Muskoxen apparently have maintained a well-fertilized*, *highly productive ecosystem*, *…”*. In Henry [19] soil moisture, but not temperature was mentioned as a factor influencing sedge meadow in the pass.

**Fig 2:**
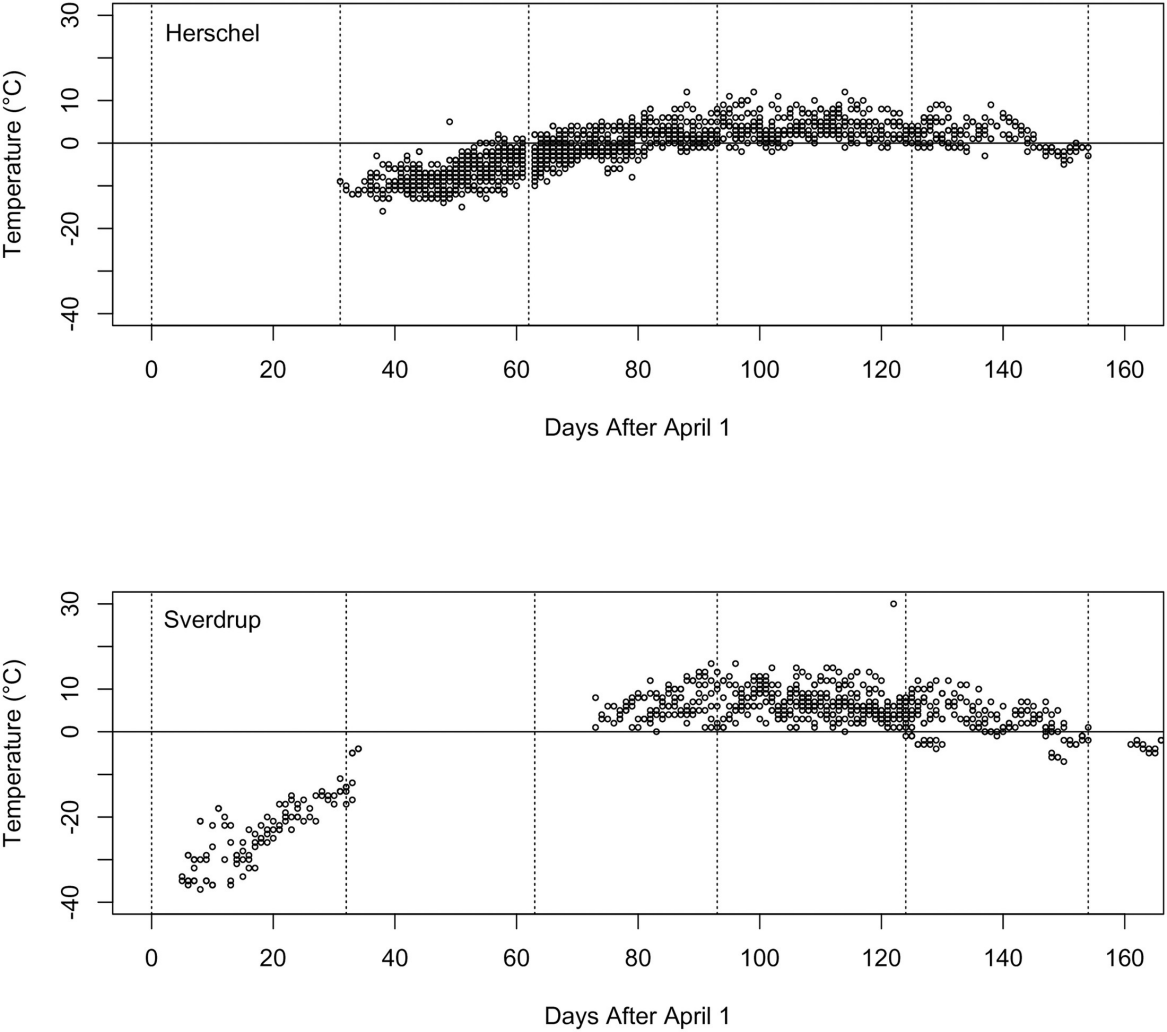
Temperature measurements from field camps in the Herschel and Sverdrup areas between the years of 1974–1993. Any measurements from the PCSP database [20] were included; see text for details.

Some data are available from the twice daily weather reports sent in from field camps over the course of the years 1974–1993 [20], and all data from all years for the two regions were plotted (Table 1, Fig 2), although there is little overlap between dates where Sverdrup Pass camps reported and when Herschel did. Although June tends to be warmer in Sverdrup Pass than on in the Herschel area, there is little discernible difference in July or August. Below zero temperatures seem to return in late August at approximately the same time and there are no data in May and early June to compare the end of the ice-cover season. So we are left with no data permitting a comparison of the sites, or an interpretation of the causes of the oasis.

In summary, another interpretation of the “oasis” sites is that they are highly productive due to continual water supply from nearby glaciers and combined with bedrock, this provides more available nutrients, in an area that may or may not be warmer than “cold”-“cool”-“warm” sites [9]. The water supply may have ensured that the sites did not occasionally dry, as happens in the lowland sites sampled by Griffiths et al. [1, 21], leading to the increased vegetation density surrounding and within the lake. During the Little Ice Age, the area may have had more extensive snow and ice and evidence suggests glaciers were more extensive in the past [10, 22], leading to the low sedimentation rate (next section). Therefore these sites may have not been historically warm.

**Fig 3:**
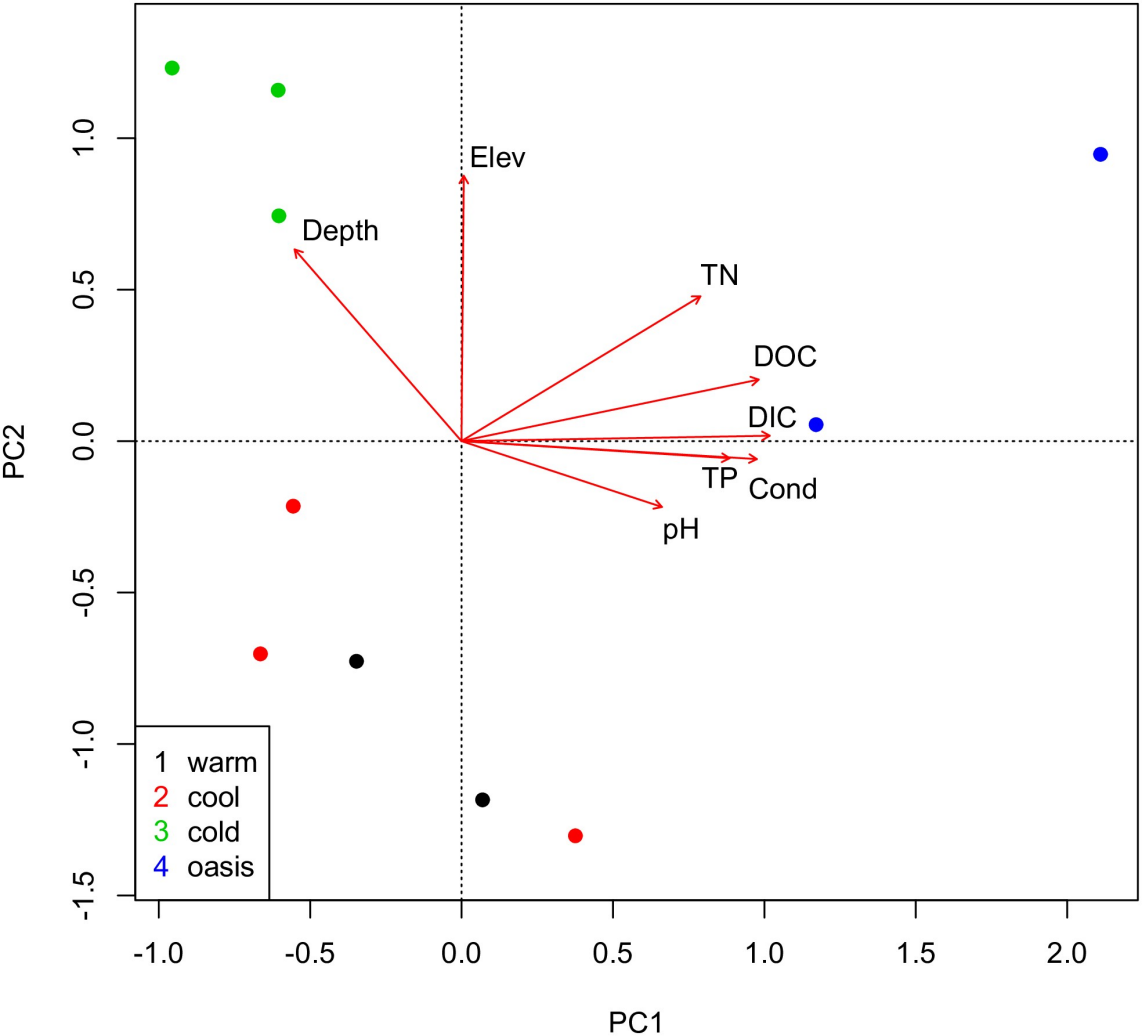
Principal components biplot of the data in Griffiths et al. (2017) Table 2. Axis 1 explains 60.1% of the variance and axis 2 explains 17.6%.

In addition to insufficient evidence of differences in ice cover, the four groups of lakes differ in depth and nutrient concentration which would affect diatom abundance and diversity. A principal components analysis the data in Table 2 of [1] (Fig 3) shows “cool” and “cold” sites overlap in characteristics and that the groups chosen by Griffiths et al. [1] are confounded with other variables. The “oasis” sites are relatively nutrient-rich with a continuous water supply, “cold” sites are deeper lakes, and “warm” and “cool” sites have low total Nitrogen, high dissolved inorganic carbon and are very shallow ponds. Even if the growing season (i.e., lack of ice cover season) were identical at all sites, these differences in physical and chemical characteristics would affect diatom assemblages and production [e.g., 23, 24].

### 2) Problems with their chronologies and lack of dating in some cores make it difficult to test hypotheses about the timing of changes in the diatom assemblages

Griffiths et al. [1] predicted that the major change in diatom communities (a shift from “condition 1” to “condition 2”) would have occurred earlier in warmer sites than in colder sites. To confirm or refute this hypothesis, it is necessary to have accurate and precise chronologies for their sequences. Dating of sediments is probably the biggest challenge to paleoecologists working in the Arctic. In the Methods, the authors acknowledge the problems in their attempts to date the cores and they do the best that they can to date the cores.

The cores are dated in three ways:

a) The six “cool” and “cold” sites had ^210^Pb ages. For ages below the levels where they could measure unsupported ^210^Pb, they extrapolated.
b) “Warm” site cores, Col Pond and Elison Lake, are undated. Instead, they used the ages of previously published cores [25] collected in 1978 from the same sites, which they visually correlate to the current cores using the diatom stratigraphy.
c) The “oasis” site SV5 has no ^210^Pb ages, so the chronology is based on one radiocarbon age; SV8 has ^210^Pb determinations and one ^14^C date.

#### a) Dating of the “cold” and “cool” sites

Griffiths et al. [1] Fig 3 presents the dating of the six cores from the “cool” and “cold” sites and these are here summarized in Table 1. Under the best conditions, ^210^Pb dates can only provide age estimates for ~150 years, but in the “cold” and “cool” sites, reliable estimation of dates is apparently restricted to the 20^th^ century and in some cases (Paradise Pond, High Lake, Proteus Lake) to the late 20^th^ century (Table 1). Forty-four percent of the assemblages are dated by extrapolation below the depth where unsupported ^210^Pb is greater than supported values (and not by interpolation; [1] Fig 5 & 6 captions).

#### b) Dating of the “warm” sites

Douglas et al. [25] presented diatom analyses from frozen cores from three sites: Col Pond, Camp Pond and Elison Lake. Camp Pond, the third pond studied by [25] was dropped for this study; it seems it has dried, as have other ponds in the area [21]. Col Pond has only ^210^Pb activity; in which unsupported ^210^Pb is restricted to the uppermost 4 cm [25] or 0.5 cm [1]. Therefore, the section of the 2011 core with unsupported ^210^Pb is much shorter than the 1994 core; although this variability in sedimentation rate is frequently observed in different cores from the same site, another possibility is disturbance of the sediments. Below this date, the age must be extrapolated. Douglas et al. [25] estimate that it covered “*at least 8000 years*”, and Griffiths et al. [1] state that it is dated by ^210^Pb and ^14^C. Douglas et al. [25] do not present any ^14^C dates for this site, instead referring to [11] for the chronology. However, Blake [11] does not present any ages for Col Pond; indeed, states (pg 1969) “*Unfortunately*, *no age determinations are available from this core*”. In this core, the upper one, or perhaps two, centimetres date to a time period when there is measurable unsupported ^210^Pb in the core, and Griffiths et al. [1] assign this to CE1850. Elison Lake does have a radiocarbon date [25] (3850 +/-100 ^14^C yr BP at 45 cm) and the uppermost 3 cm contain unsupported ^210^Pb, assigned to CE1850.

However, in the other cores presented in Fig 3 of Griffiths et al. [1], the unsupported ^210^Pb is restricted to the 20^th^ century (Table 1). Douglas et al. [25] did not present an age model for Col Pond or Elison Lake cores, simply assuming that the unsupported ^210^Pb extended back to 1850CE. Given this new information about deposition of ^210^Pb from this region, in ponds that seem to be comparable, it would seem that the decision to assign 1850CE to this transition should be revisited. Given the ambiguities in correlating the cores of Griffiths et al. [1] to those of Douglas et al [25] and the lack of dates of the Col Pond or Elison Lake cores, conclusions about the timing of the changes in these two sites are not supported by the data.

#### c) Dating of the “oasis” lakes

In the Sverdrup Pass ponds, SV8 and SV5, dates of 2000–3000 years were obtained at ~10 cm. In Fig 7b of [1], they date 6 cm at CE1874, and 10 cm at ~3064 cal yr BP. This leaves approximately 3000 years in 4 cm followed by an accumulation of ~140 years in 6 cm. But there are no transitions in the diatom assemblages in spite of such a large change in accumulation. Biological production is an important component of total sediment accumulation, and this implies a significant change in sedimentation but no change in the diatom communities. But if these are such productive systems, where did the sediment go? There are a number of possibilities:

1. There is little accumulation, for example the organic material is being constantly decomposed or recycled (during the very short growing season, because the ponds freeze solid in winter). However, productive sites typically accumulate larger amounts of sediment than unproductive sites. If this option is accepted, where did the accumulation go?
2. The dates are too old. The description of the material sampled says it is “woody, herbaceous stems”. Was this on a soil, or were the stems transported into the lake? Did the plants die, and they were blown onto the lake, after sitting on the landscape for a long period of time? This is certainly a possibility, and is thought to occur in Arctic systems [26]. However, with only one date, it is difficult to make inferences about this possibility. If the sequence were longer, and there were more dates, then it might be possible to hypothesize about the missing sediment.
3. There is a hiatus in the core. This is a possibility; indeed, SV8 is less than a kilometer from a glacier that has apparently been retreating since the LIA and less than 0.5 km from the boulder field created from the retreating glacier [22]. It is therefore possible that it was continuously frozen for substantial periods of time and not historically warm.
4. In these extremely shallow systems, the mixing of the water during the frequent windy periods churns up the sediment, which is constantly disturbed, re-suspended and removed from the system. This is a likely possibility.

These options, occurring in isolation or together, would compromise temporal inferences of past conditions, especially to test hypotheses requiring precise chronologies.

### 3) The interpretation of changes in the diatom assemblages and production in these short cores are contradicted by longer sequences from the same area. In addition, diagenesis or mixing of the uppermost sediments makes it difficult to draw conclusions from such extremely short sediment sequences

Griffiths et al. [1] summarize the changes in the diatoms into two zones using a stratigraphically-constrained cluster analysis, which they call “Condition 1” and “Condition 2” and which are considered to be high-diversity and low-diversity respectively. They also summarize the diatom changes with a measure of richness and total production using chlorophyll *a* (Chla) measured in the sediment. Griffiths et al. [1] do not, however, measure diatom concentrations or accumulation rates in the sediments, which would enable the analysis of the association of production and diversity. Biogenic silica would be another option.

In the present study, the basic results are as follows.

(a) In the “warm” and “cool” sites, Chla was below detection in deeper sediments and increased in the uppermost 1–3 cm.
(b) In the “oasis” sites, Chla was above detection in deeper sediments and increased in the uppermost 3–5 cm.
(c) In West Lake, the deepest lake in their study sites, Chla remained above detection throughout, with some variations. In Proteus and High Lakes, the increase in the uppermost centimeter was actually a return to values seen below 3–4 cm; this is not plotted in Fig 8 of [1], which is truncated at 1700CE, but included in the data files (S1 Data), in contradiction to their conclusion from the Discussion (p. 17) that *“Two of our “cold” sites*, *High Lake and Proteus Lake*, *record profiles that are consistent with this hypothesis*, *as production remains stable until the most recent intervals”*.

The curves of Chla sometimes broadly parallel those of N2 (Elison Lake, Col Pond, High Lake) and sometimes do not (the other sites). Although Griffiths et al. [1] associate these changes in primary production with changes in the assemblages as summarized in conditions 1 and 2, the association is not clear enough to accept or reject their hypotheses. Again, the extreme shortness of the data records does not permit Griffiths et al. [1] to place these changes in any historical context.

Griffiths et al. [1] simplify the diatom changes by dividing the assemblages into two groups using a constrained cluster analysis. Several aspects of the application of the cluster analysis should be noted. A cluster analysis will always divide a biostratigraphic sequence into zones, even if there are no changes in the data. In a continually changing time series, a cluster analysis will place zones at some location, depending on the method used for zonation, and this will hide the fact that the changes are slow and continuous. Since they use a stratigraphically-constrained method, if a site were oscillating between two “conditions”, each subsequent one would be considered as a separate zone and not as a return to a previous state, as is occurring in West Lake (S1 Data). Based on their own results (supplemental files), I would in some instances locate the zones in different places (S1 Data); it is not always clear how Griffiths et al. [1] interpret the dendrograms. Finally, in some sites, very small changes are causing the analysis to divide the sequences into zones. This methodology therefore makes it difficult to use the timing of the transition from condition 1 to condition 2 between sites as evidence supporting their hypothesis.

The “cold” sites have remained in condition 1, and the “oasis” sites in condition 2, according to the interpretation of Griffiths et al. [1]. In the “cold” sites they saw no aquatic vegetation and that this would contribute to these sites remaining in condition 1, but these sites are deeper lakes. The disturbance during ice melt, every year irrespective of length of season, would make vegetation establishment in the nearshore and shallow portions of the lake difficult, as mosses or diatoms would need to be able to colonize every year; this helps explain the presence of disturbance diatom species.

At High Lake and Proteus Lake, they show there is a shift in the upper sediments to condition 2, but it is not based on the cluster analysis. They place a condition 2 zone on the uppermost sample of High Lake, but the cluster analysis divides the diagram into 3 zones, with the breaks located between 1.5–1.75 and 3–3.25 cm. Proteus Lake also has a zone boundary between 4.25 and 4.5 cm, coincident with Chla decreasing to below detection. The earlier period of high Chla does not coincide with an increase in epiphytic taxa.

In the “warm” and “cool” sites (the shallow ponds in the lowlands) there was no detectable Chla in deeper sediments, and small increases in the upper sediments. Diatoms were apparently being deposited in the sediments, but no Chla accumulated. Large changes in diatom concentrations have been recorded in Holocene diatom studies from the Canadian Arctic, and these may be associated either with climate changes, with lake history, or with diagenesis [27]. Low diatom concentrations in sediments seem to be associated with warm temperatures at many sites, perhaps due to dissolution of the diatoms [27]. Without data on diatom concentrations, it is difficult to make sense of production history of these short sequences. There is no information given about the nature of the sediment in these sequences, such as loss-on-ignition (LOI) or grainsize, which might indicate something about the sedimentation history of the sites.

Arctic sediment cores frequently have a more-organic surface layer, underlain by clayey inorganic grey sediment below (personal observation). Griffiths et al. [1] apparently interpret this as response to a longer growing season due to global warming. However, in the early Holocene, when the Canadian High Arctic was as warm or warmer than present [28–30], the sediment remained inorganic and clayey e.g., [26, 31, 32, 36]. An interpretation of the diatom records of Griffiths et al. [1] is that the upper layer is the recently deposited mixed sediment that has not yet been incorporated into the permafrost and has not yet undergone diagenesis. Indeed, Blake [33] has shown that in many shallow ponds, no sediment accumulates, suggesting the shallow sediments reported by Griffiths et al. [1] could be simply a thin surface organic layer. The lack of variability in the deeper sediments of the “cold”, “cool” and “oasis” sites could be due to a number of sources, including diagenesis, mixing or smearing along the edge of the corer. With only percentage data, even if there were almost no diatoms, or many, the percentages would look the same. The decrease to negligible levels below the surface layers cannot be interpreted in climatic terms, or due to ice cover.

Griffiths et al. [1] interpret the diatoms in the uppermost sediments of West Lake as showing no change in association with recent climate changes. However, a Holocene diatom record is available for this site [34], and Griffiths et al. [1] completely change the interpretation of [34]. West Lake did show large changes in the diatoms in the past, for example, a nearly complete replacement of an early Holocene flora with another. Rouillard et al. [34] associate these changes with both climate and watershed changes.

One of the major conclusions of Rouillard et al. [34] is that West Lake has shown a significant response to 20^th^ century global warming, interpreted through an increase in *Cyclotella radiosa*: “*This shift of the last century is the most ecologically unique in the Holocene record and is indicative of longer ice-free summers consistent with modern climate warming*.” [34; abstract]. This conclusion is in disagreement with Griffiths et al. [1], who interpret no change during the same time period. Does this mean that an increase in *C*. *radiosa* does not indicate global warming [35] or only in this site? Why was West Lake so “sensitive” to past climate changes but not to the present one? Of course, new data can cause re-evaluation of previous hypotheses, but it seems that such a major reinterpretation of the West Lake data should have been addressed and justified by Griffiths et al. [1].

Holocene records are also available for Col and Elison in [25] and here the interpretation is reversed. In Douglas et al. [25] and in Griffiths et al. [1], they concluded that Col and Elison showed no changes through the Holocene, until the past 150 years. The early to mid Holocene was, by most reconstructions, as warm as or warmer than present in the Canadian Arctic, depending on region [29, 30]. According to the interpretation of Griffiths et al. [1], Douglas et al [25] and Rouillard et al. [34] this affected West Lake but not Col Pond or Elison Lake. The interpretation of the short sequences of Griffiths et al. [1] in the context of Holocene changes is thus not clear and seems contradictory.

## Conclusions

The above discussion suggests that there is little basis for Griffiths et al.’s [1] separation of groups due to ice cover and that the physico-chemical nature of the study sites may provide a more parsimonious explanation of the diatom data. Consideration of the chemistry of the water and components of the geography of the lakes provides an alternate explanation of the four groups. A major issue in the interpretation of their study is that it based on exceedingly short, poorly-dated sequences, as short as 5 cm in some cases. The value of paleoenvironmental studies is that they can provide some historical context to interpret current changes, but the conclusions based in Griffiths et al. [1] contradict those based on previously-published complete Holocene sequences [25; 34]. Peros et al. [36] provide another example where analysis of a Holocene sequence causes re-evaluation of the conclusions based on short sequences of the past couple of centuries.

Their reliance on percentages of the diatom assemblages rather than concentrations or accumulation rates makes it difficult to identify changes in production or potential diagenesis. The use of small, very shallow ponds, which suffer from mixing of the water column and sediment, places limits on the inference possible from their cores. Ponds of 1–2 meters depth (e.g., Paradise, Elison) also suffer from disturbance. Given the almost complete lack of production in older sediments, it is simply not clear what is happening in these sequences, especially the “cold” and “warm” sites.

Therefore, an alternative interpretation of the sequences is as follows:

1. Lakes (their “cold” sites) have different diatom assemblages from ponds. Nutrient-rich ponds (their “oasis” sites) have different diatom assemblages because nutrient input depends on local conditions of bedrock or water availability, for example groundwater seepage from glacier outflow. The “oasis” sites, with high nutrients and perhaps more constant water supply, have higher diversity relative to the other sites.
2. Griffiths et al. [1] report that the diatom assemblages of West Lake showed “*no directional change*” during the past century. In contrast, a previous study of the same lake [34] interpreted the diatom changes of this same time period as showing “*longer ice-free summers consistent with modern climate warming*.”. These contradictory interpretations confuse the interpretation of “cold” sites. The sequences from the two other lakes (“cold” sites) are too short to interpret.
3. The remaining “cool” and “warm” sites, which, in fact, cannot be distinguished, are shallow ponds whose history is not clear. Interpretation of chronologies in short sediment cores from small and shallow ponds need to consider sediment mixing and alteration due to drying and refilling of ephemeral ponds. These sites are problematic for developing a paleoecological record.

## Supporting information

S1 Data Summary of the diatom data supplied by Griffiths et al. [1]. For each site, plots of N2 and Chlorophyll-a from data tables (10.5061/dryad.g7h7n). The diatom assemblage changes are summarized as the scores of the first two principal components (based on a correlation matrix and computed in C2 [2]); scores are plotted on the stratigraphic plots, and biplots, along with the variance explained by each analysis show the loadings. For each stratigraphic diagram, the zones as interpreted by Griffiths et al. [1] (leftmost of the two columns) and my interpretation (right column) are shown. See supplemental information of Griffiths et al. [1] for cluster dendrograms and broken-stick result.
(PDF)
