# Supplementary figures and images for: Multiple drivers of ecological change in Arctic lakes and ponds
Source: PLoS One. 2021 Jul 30;16(7):e0254257. doi: 10.1371/journal.pone.0254257 (PMC8324333; doi:10.1371/journal.pone.0254257)

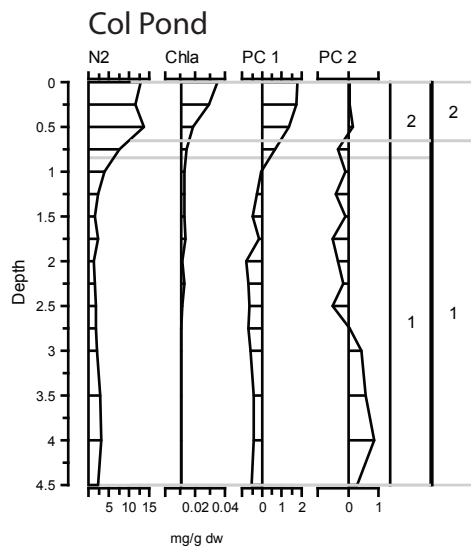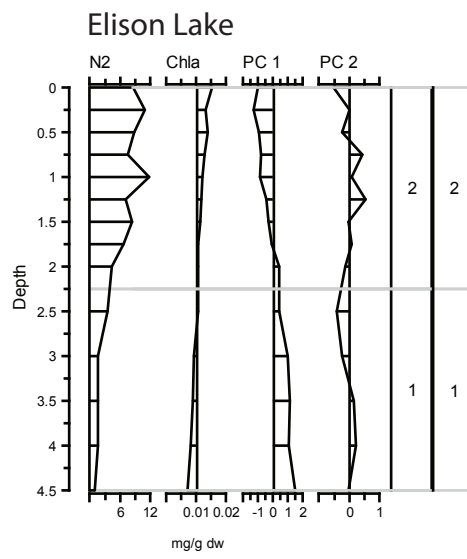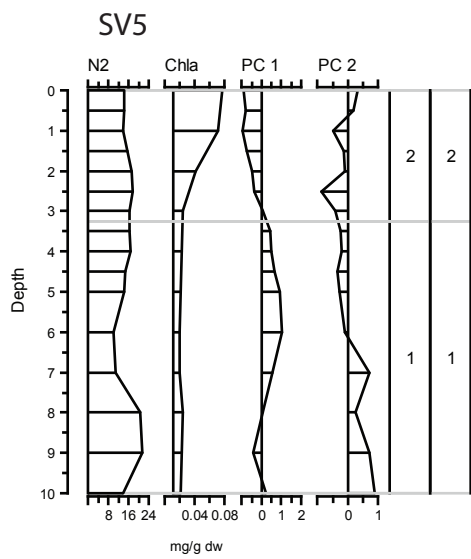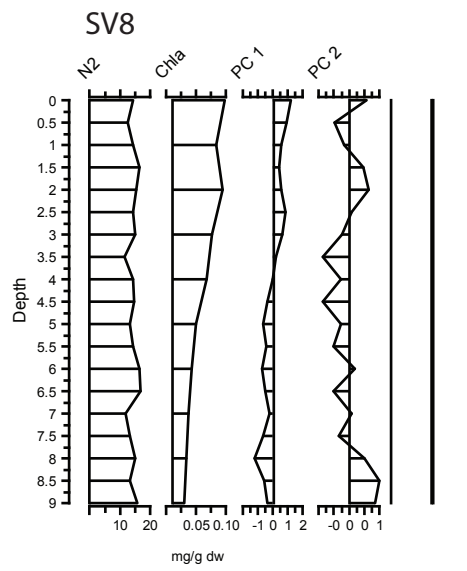

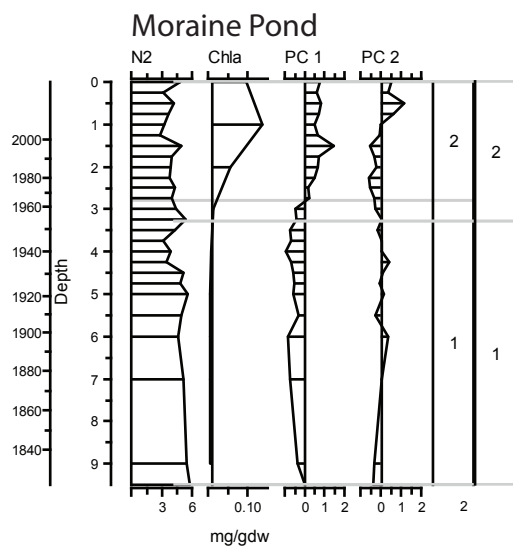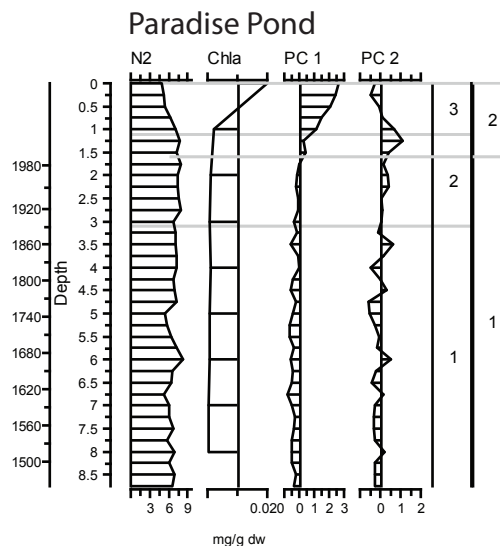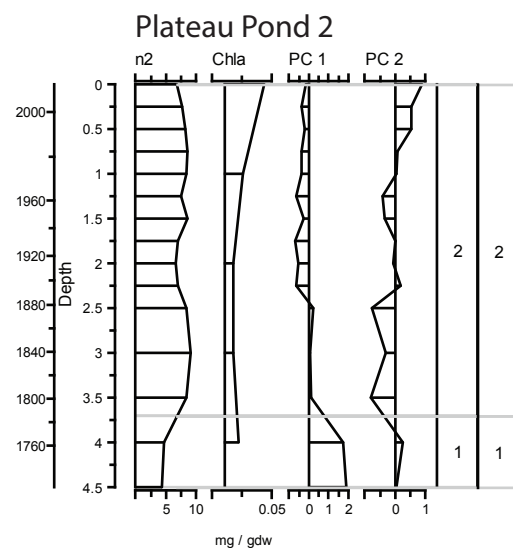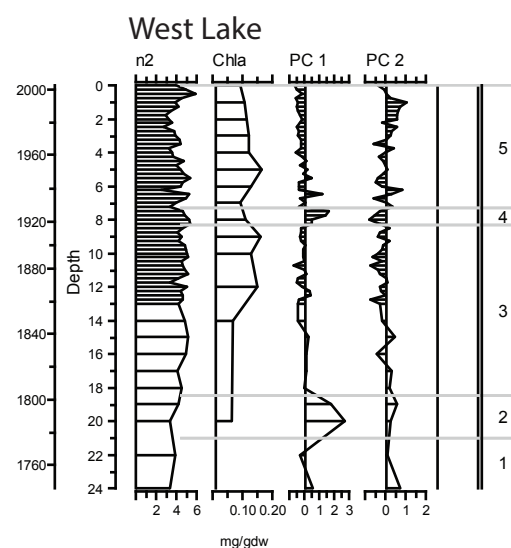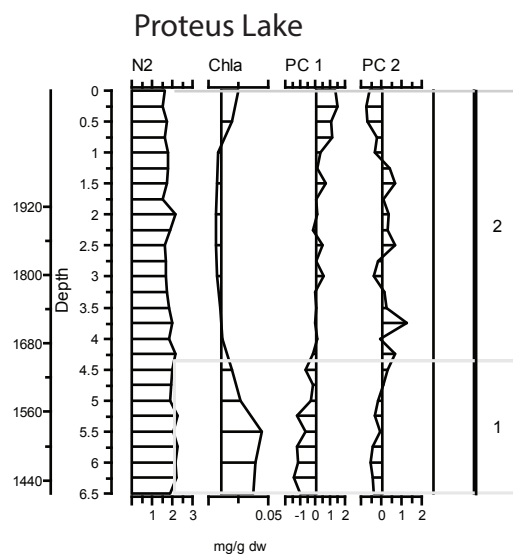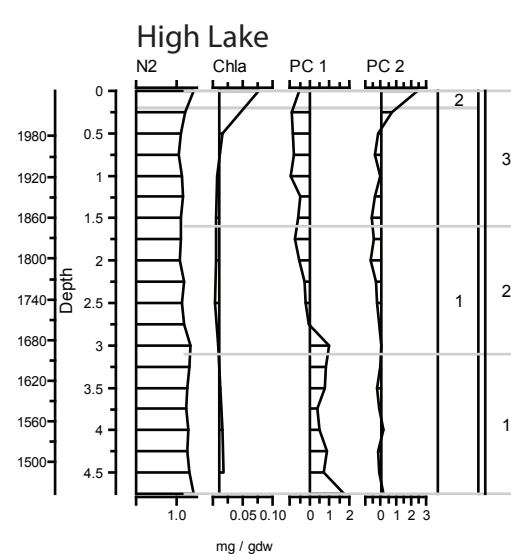



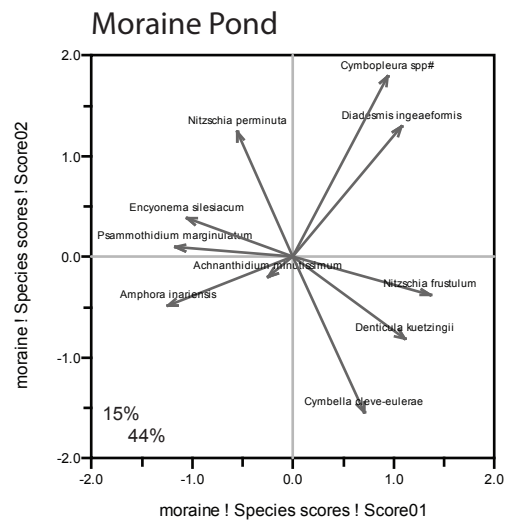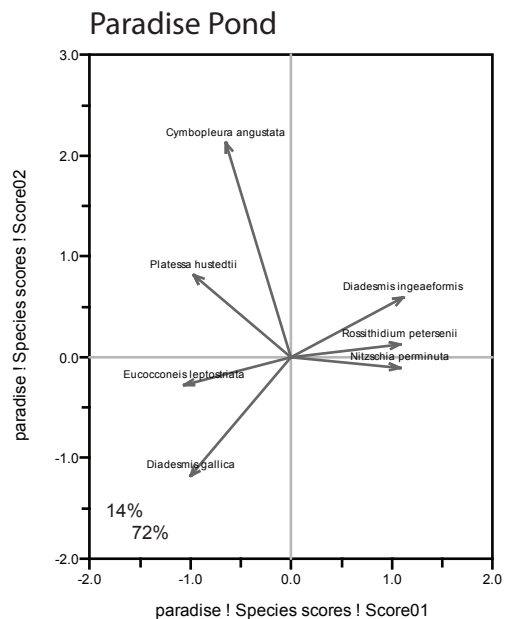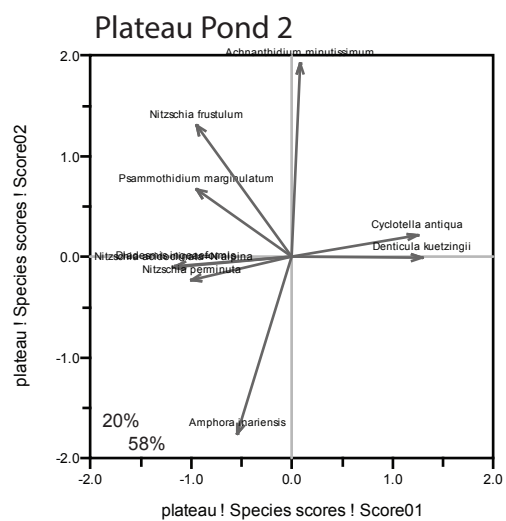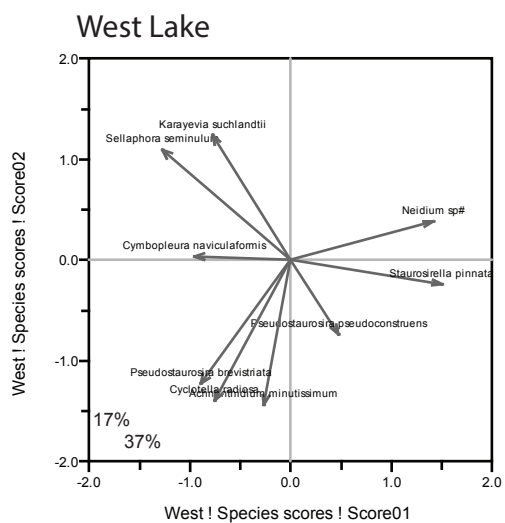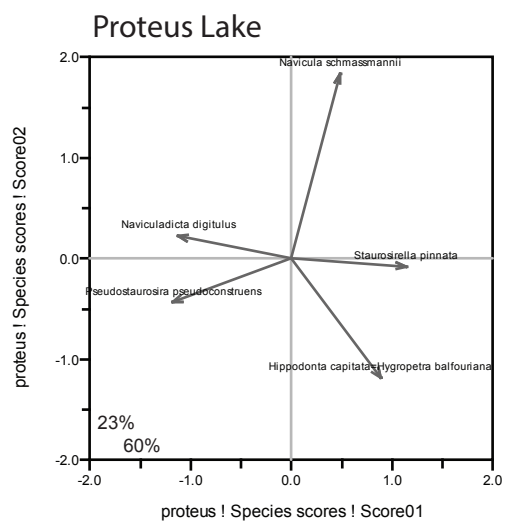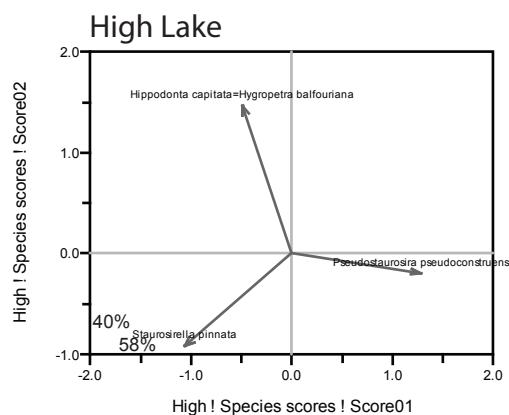

Supplement: S1 Data — For each site, plots of N2 and Chlorophyll-a from data tables (10.5061/dryad.g7h7n). The diatom assemblage changes are summarized as the scores of the first two principal components (based on a correlation matrix and computed in C2 [2]); scores are plotted on the stratigraphic plots, and biplots, along with the variance explained by each analysis show the loadings. For each stratigraphic diagram, the zones as interpreted by Griffiths et al. [1] (leftmost of the two columns) and my interpretation (right column) are shown. See supplemental information of Griffiths et al. [1] for cluster dendrograms and broken-stick result. (PDF) [file pone.0254257.s001.pdf]
